# The Role of Residential Early Parenting Services in Increasing Parenting Confidence in Mothers with A History of Infertility 

**DOI:** 10.22074/ijfs.2016.4907

**Published:** 2016-06-01

**Authors:** Marjan Khajehei, Lynette Finch

**Affiliations:** 1 Research Coordinator, Karitane, Carramar, NSW, Australia; 2 Conjoint Lecturer, University of New South Wales, Sydney, Australia; 3 Residential Family Care Unit, Carramar, NSW, Australia

**Keywords:** Infertility, Postnatal, Parenting Confidence, Residential Units, Parenting Services

## Abstract

**Background:**

Mothers with a history of infertility may experience parenting difficulties and challenges. This study was conducted to investigate the role of residential early parenting services in increasing parenting confidence in mothers with a history of infertility.

**Materials and Methods:**

This was a retrospective chart review study using the quantitative data from the clients attending the Karitane Residential Units and Parenting Services (known as Karitane RUs) during 2013. Parenting confidence (using Karitane Parenting Confidence Scale-KPCS), depression, demographics, reproductive and medical history, as well as child’s information were assessed from a sample of 27 mothers who had a history of infertility and who attended the Karitane RUs for support and assistance. The data were analyzed using SPSS version 19.

**Results:**

More than half of the women (59.3%) reported a relatively low level of parenting confidence on the day of admission. The rate of low parenting confidence, however, dropped to 22.2% after receiving 4-5 days support and training in the Karitane RUs. The mean score of the KPCS increased from 36.9 ± 5.6 before the intervention to 41.1 ± 3.4 after the intervention, indicating an improvement in the parenting confidence of the mothers after attending the Karitane RUs (P<0.0001). No statistically significant association was found between maternal low parenting confidence with parental demographics (including age, country of birth, and employment status), a history of help-seeking, symptoms of depression, as well as child’s information [including gender, age, siblings, diagnosis of gastroesophageal reflux disease (GORD) and use of medication].

**Conclusion:**

Having a child after a period of infertility can be a stressful experience for some mothers. This can result in low parenting confidence and affect parent-child attachment. Our findings emphasized on the role of the residential early parenting services in promoting the level of parenting confidence and highlighted the need for early recognition and referral of the mothers with a history of infertility to such centers.

## Introduction

With the improvement of assisted reproduction technologies (ART) and public awareness, there is an increasing rate of children born to infertile parents (1). Having a baby after shortor long-term infertility can be joyful to all parents. This form of transition to parenthood may be most likely to be considered as a challenging experience for some parents, especially for mothers during early parenthood (2). Some women with a history of infertility experience less severe symptoms of postnatal mental health problems and easily adjust to the changes, which is likely due to their socioeconomic advantages and supportive partners (3). Nevertheless, different studies have reported higher levels of postnatal depression in infertile women as they underwent infertility treatment, experienced obstetric complications associated with ART pregnancy and had idealistic expectations about their parenting role (3,5). As compared with women without a history of infertility, another study has showed that infertile women conceiving through ART experienced greater level of psychological disorders such as distress, over-protectiveness, fatigue, anxiety, depression, as well as inability to develop parenting skills (6). Furthermore, feelings of guilt or shame may affect the mother’s mental health, relationship with the partner, parenting skills, parent-child relationship and child’s development (3,5). 

Different studies have also shown the association of early parenting difficulties with negative birth experiences, parental physical and mental health problems, sleep deprivation, distress, mental and physical disabilities of the child, insufficient family-child interactions, inadequate support from partner, family and friends (7,8), intimate partner violence, as well as divorce (9,10). Another research has revealed that 20-30% of mothers referred to the parenting centers met the diagnostic criteria for major depression, anxiety disorder and severe fatigue. These negative experiences have been reported to affect mental, emotional, physical and social development of the child (11). It is becoming increasingly evident that the infant’s brain development is shaped by the positive and negative experiences of early parenting (12). New evidence suggests that the infant’s unsettled behaviour is associated with the maternal characteristics and psychosocial environment (13). These indicate the importance of early intervention in cases when the parenting experiences are less favourable (12). 

## Karitane Residential Units and Parenting Services

In order to facilitate a smooth transition to parenthood and minimize its undesirable effects on the parents’ well-being and child’s development, the Karitane Residential Units and Parenting Services (known as Karitane RUs) have been established in New South Wales (NSW), Australia, under the name of the Australian Mother Craft Society in 1923. These centers aim to provide sufficient parenting support to families struggling with the demands of parenting, help them promote their knowledge and skills, as well as enhance their confidence in raising their children. The families (parents and their children) reside in these centers for a limited period of time (3-5 days). Their parenting skills and children’s behaviours are observed, and sufficient advice and support are provided on a case by case basis. The Karitane RUs, as tertiary level public hospitals, provide services including psychology services, day stay, residential services and outreach services to families in order to promote children’s wellbeing and family-child emotional attachment. They are staffed by a team of child and family health nurses, parent-craft/mother-craft nurses, general practitioners, pediatricians, psychiatrists, psychologists and social workers. Parents are commonly referred to the Karitane RUs for the following reasons: establishment of a bedtime routine, feeding problems and struggling with managing unsettled infants. In order to provide the most effective and high quality services, the professionals work with parents through a partnership approach. Through this model of care, the clinicians work as facilitators assisting the parents to promote their problem-solving skills, self-esteem, self-efficacy and confidence. 

Parenting confidence is a feeling of competence in their parenting role. The partnership-approach focuses on the development of relationship, encouragement of the parents to choose the best intervention that works for the family and improvement of their parenting confidence (13,14). 

Assessment of parenting confidence in the mothers with a history of infertility can provide better knowledge about how infertility can affect early parenting behaviours of these women. To the knowledge of the authors, there is a lack of research on the parenting confidence of the mothers with a history of infertility who receive support and education in the parenting centers. Thus, this study aimed to evaluate the effect of residential early parenting services on the parenting confidence of the mothers with a history of infertility during the first postpartum year. We hypothesized that attending these centers can improve parenting confidence of these mothers. The study also intended to investigate the factors associated with low parenting confidence. 

## Materials and Methods

This was a retrospective chart review study using the quantitative data from the clients’ records of the Karitane RUs during 2013. The study was part of a larger project in which 144 records of the mothers with infants of 0-12 months were randomly selected. Among them, 27 mothers with a history of infertility attended Karitane RUs for support and assistance. The data of these entire 27 mothers were considered for further evaluations. 

The project was approved by the South Western Sydney Local Health District (SWSLHD) Human Research Ethics Committee (HREC) prior to its commencement. Since this was a retrospective study using the previously recorded data of the clients’ files and the results of the study were de-identified and analyzed collectively, we were not required by the SWSLHD HREC to obtain an informed consent from clients to analyze the data and publish the results. 

### Karitane Parenting Confidence Scale

The Karitane Parenting Confidence Scale (KPCS) as a validated 15-item self-report measure was developed to assess parental confidence in caring for their child. The internal consistency (Cronbach’s alpha=0.81), test-retest reliability (r=0.88), and discriminant and convergent validity of the KPCS have already been documented in a literature (15). Every item on the KPCS is scored from 0 to 3. After summing the points, the total score can range from 0 to 45. The cut-off score for the KPCS is 39 or less, indicating clinically significant low levels of parenting confidence. 

### Edinburgh Postnatal Depression Scale

The Edinburgh Postnatal Depression Scale (EPDS) as a validated 10 item self-report questionnaire was designed to screen and identify mothers for symptoms of depression during pregnancy and after childbirth (16). Each item is scored from 0 to 3, while items 3 and 5-10 are scored reversely. The total score varies from 0 to 30. A score between 10 and 12 indicates probable minor depression, and a score greater than 12 indicates probable major depression. 

### Additional data collected from clients’ files

After a comprehensive review of the literature, the following variables were extracted from the clients’ records: history of infertility, KPCS and EPDS scores on both admission and discharge, parental demographics (age, education, career, marital status, residence, country of birth, and main language spoken at home), obstetrics and gynaecological history, medical history, child’s information [gender, age, siblings, diagnosis of gastroesophageal reflux disease (GORD), and use of medication], history of mental health problems, domestic violence, alcohol use, cigarette smoking, and history of parenting intervention. 

### Demographics of the families referred to the Karitane Residential Units

The following inclusion criteria were used to refer families to the Karitane RUs: unable to establish bedtime routines, feeding problems, and difficulties in parent-child relationship, while the exclusion criteria was mothers with serious mental illness. The clients referred to the Karitane RUs by a health care professional were contacted by a clinical nurse who explained the admission process and procedure. After collecting relevant information over the phone and setting an admission date, an admission package was sent to the clients. On the admission day, the clients with their children attended the Karitane RUs and completed relevant forms. They also completed the EPDS and the KPCS before and after (on day four of their stay) the intervention. Then education, support and guidance in an intensive program were provided for parents by a multidisciplinary team consisting of nurses and allied health staff on an inpatient basis. The details of the service in the Karitane RUs were documented in the earlier publications (10,17). 

### Service summary in Karitane Residential Units

A summary of the day-by-day care provided to the families in the Karitane RUs is as follows: 

#### Monday:

After admission, all parents were required to sign a consent form and complete the measurement tools (EPDS and KPCS), while the mothers were screened for domestic violence. The children were also examined by a pediatrician. If they took certain medications, a management plan would be prepared according to the findings at the time of assessment and their medications would be reviewed. Reason for admission and goals were identified during the comprehensive assessment, and a family management plan (including strategies) was discussed in partnership with the parents on a caseby-case basis. These strategies were implemented on Monday afternoon. 

#### Tuesday: 

The mothers made appointments with allied health professionals if needed. The discussion about how to prepare formula for the infants, also known as “Formula Preparation Talk”, was held in the food room. A pram-walking group supervised by a clinician and a playroom coordinator was done either on Tuesday or Wednesday dependent on the weather conditions. Otherwise, a DVD on effective parenting was played for parents. The mothers attended both the toddler and relaxation groups in the afternoon. 

#### Wednesday:

Mothers were offered massages.Adjustment to parenthood group was held in order to encourage the parents to practice independently, support given as required. On Wednesday evening, a group, called Ready, Set, Go, was held to prepare the mothers for the discharge where they could ask their questions and find out what to expect at home. Father group was held at night. 

#### Thursday:

Clinicians strongly encouraged parents to use the different strategies trained more independently, support given as required. Relevant forms and documents were also completed. Discharge summary, including plans for discharge, follow-ups and appointments, was completed and finalized in partnership with a clinician. Those parents who stayed in the RUs until Friday attended the relaxation group and watched movie at night along with other parents. 

#### Friday:

Mothers completed an exit survey and left the residential units with their children by 10.30 am. 

### Data analysis

The quantitative data were analyzed by Statistical Package for Social Science software (SPSS, SPSS Inc., USA) version 19.0. The data validation occurred prior to the data entry, during the data entry, and post data entry as follows: visual review, value range checks, field type checks, and logical checks. Descriptive statistics were calculated for the variables of interest. Chi-square test (χ2) (Fisher’s exact test if applicable) was used to investigate the frequency distribution of all variables between the clients with low and high levels of parenting confidence. Paired t test was carried out to investigate the difference in the scores of the KPCS before and after the intervention. To test our hypothesis, we used independent samples t test to measure the differences in the parenting confidence and symptoms of depression at 0-6 months and 7-12 months postpartum. A P value less than 0.05 was considered to be statistically significant. 

### Results

Table 1 shows that 14.8% of the mothers reported symptoms of depression on admission to the Karitane RUs, and 59.3% reported low level of parenting confidence.

**Table 1:** Higher Rate of the depression and low level of parenting confidence in the mothers with a history of infertility


	n	%

Symptoms of Depression		
Non-depressed	23	85.2
Depressed	4	14.8
Parenting confidence before the intervention		
Low level of parenting confidence	16	59.3
High level of parenting confidence	11	40.7
Parenting confidence after the intervention		
Low level of parenting confidence	6	22.2
High level of parenting confidence	21	77.8


The rate of low parenting confidence, however, dropped to 22.2% after receiving 4-5 days support and training. Results of the Chi- square test revealed no statistically significant association between the low parenting confidence with maternal age, education, career, country of birth, level of support, depression, recent major stressor, parity, antenatal and postnatal health complications, mode of delivery, as well as child’ information (includ- ing gender, age, siblings, diagnosis of GORD and use of medication) (P>0.05, Table 2). Results of the paired t test showed that the mean score on the KPCS increased from 36.9 ± 5.6 before the intervention to 41.1 ± 3.4 after the intervention, indicating an improvement in the parenting confidence of the mothers after attending the Karitane RUs, which was statistically significant (P<0.0001). This was also confirmed by a large effect size [degree of freedom (df)=0.905], indicating clinically meaningful changes in the parenting confidence of the mothers with a history of infertility (Table 3). 

**Table 2:** Parenting confidence level and demographics of the mothers with a history of infertility on admission to Karitane RUs using chi-square test


	Parenting confidence on admission		P value
	Low level of confidence^*^ (n=16)	High level of confidence^*^ (n=11)	

Age group (ranged from 20 to 45)			
20-29	7 (43.7%)	6 (54.5%)	0.778
30-45	9 (56.2%)	5 (45.5%)	
Country of birth			
Australia	10 (62.5%)	10 (90.9%)	0.183
Other	6 (37.5%)	1 (9.1%)	
Education			
No formal education	0	1 (9.1%)	0.402
Diploma or lower	4 (25%)	1 (9.1%)	
Tertiary education	5 (31.2%)	5 (45.5%)	
Unknown	7 (43.7%)	4 (36.4%)	
Career			
Not employed	1 (6.3%)	2 (18.2%)	0.059
On maternity leave	1 (6.3%)	4 (36.4%)	
Currently working	14 (87.5%)	5 (45.5%)	
Support			
Partner only	3 (18.8%)	3 (27.3%)	0.662
Partner/Family/Friend/Clinicians	13 (81.3%)	8 (72.7%)	
Depression			
Non-depressed	13 (81.3%)	10 (90.9%)	0.624
Depressed	3 (18.8%)	1 (9.1%)	
Recent major stressors			
No	7 (43.7%)	5 (45.5%)	1
Yes	9 (56.2%)	6 (54.5%)	
Parity			
Primiparous	8 (50%)	6 (54.5%)	1
Multiparous	8 (50%)	5 (45.5%)	
Antenatal health complications			
No	13 (81.3%)	8 (72.7%)	0.662
Yes	3 (18.7%)	3(27.3%)	
Postnatal health complications			
No	14 (87.5%)	10 (90.9%)	1
Yes	2 (12.5%)	1 (9.1%)	
Mode of delivery			
Vaginal delivery(spontaneous or assisted)	12 (75%)	8 (72.7%)	1
Caesarean section (C/S)	4 (25%)	3 (27.3%)	
Child			
Age			
0-6 months old	12 (75%)	4 (36.4%)	0.061
7-12 months old	4 (25%)	7 (63.6%)	
GORD			
No	10 (62.5%)	10 (90.9%)	0.183
Yes	6 (37.5%)	1 (9.1%)	
Use of medication			
No	9 (56.3%)	9 (81.8%)	0.231
Yes	7 (43.8%)	2 (18.2%)	

Karitane RUs; Karitane Residential Units, GORD; Gastroesophageal reflux disease, and ^*^; Values are given as number (%).

**Table 3:** Mean scores of the KPCS before and after the intervention in the mothers with a history of infertility using paired t test


	Before the intervention Mean ± SD	After the intervention Mean ± SD	t (df=26)	d	P value

Parenting confidence (KPCS)	36.9 ± 5.6	41.1 ± 3.4	5.392	0.905	<0.0001


KPCS; Karitane Parenting Confidence Scale, df; Degree of freedom, d; (Effect size)≥0.08 or higher shows a large effect, P<0.05.

**Table 4:** Scores of KPCS and EPDS at 0-6 months and 7-12 months after childbirth in the mothers with a history of infertility using inde- pendent samples t test


Time	0-6 months after childbirth (n=16)		7-12 months after childbirth (n=11)		t (df=25)	P value
Scale	Mean	SD	Mean	SD				

EPDS	7.4	4.64	5.1	4.44	1.261	0.219
KPCS before the intervention	35.2	6.08	39.4	3.83	-2.026	0.054


KPCS; Karitane Parenting Confidence Scale, EPDS; Edinburgh Postnatal Depression Scale and df; Degree of freedom.

The mean scores of the KPCS and EPDS at 0-6 and 7-12 months postpartum were calculated using independent samples t test. It was shown that the mothers were less likely to have symptoms of depression at 7-12 months postpartum compared with 0-6 months after childbirth (5.1 ± 4.4 vs. 7.4 ± 4.6, respectively). Nevertheless, no statistically significant difference was found between the two time points (P=0.219). Similar results were shown for the mean score of the KPCS, indicating lower level of parenting confidence at 0-6 month postpartum compared with 7-12 months after childbirth (39.4 ± 3.8 vs. 35.2 ± 6.08, respectively). However, the difference between the two time points was not statistically significant (P=0.054) (Table 4). 

## Discussion

This study is the first to demonstrate the risk factors of low parenting confidence in the mothers with a history of infertility and the impact of residential early parenting service on their parenting confidence. A research has shown that the prevalence of cry-fuss problems decreases from 19.1% at 2 months to 5.6% at 4 months after birth. In addition, the prevalence rate of sleep problems decreases from 21.2% at 8 months to 16.2% at 12 months (18). It has also been reported that the unsettled infant behaviour is associated with poor parental maternal health (18,19) and less sense of competence and self-efficacy (20). Similarly, our findings showed that the parenting confidence and depression of the mothers with a history of infertility were affected by the infant’s age as they are more likely to display unsettled behaviours during 0-6 months of age. Our study also demonstrated that 59.3% of the women reported low level of parenting confidence. This finding supports the results of the study by Pearlman, in which the women with a history of prolong infertility were less confident in their parenting skills and unable to establish a bedtime routine for their children (21). Similarly, an earlier study by Gibson et al. (22) demonstrated that IVF mothers reported lower level of self-esteem and less parenting competence than the control group, while they perceived that their children were more special and unique. We found no data on the level of parenting confidence of the mothers with a history of infertility who did not attend the Karitane RUs in order to compare their related level with those who attended the RUs. Notwithstanding, a previous research has shown that women conceiving through ART were at the risk of early parenting difficulties and likely to seek support and advice from professionals. The study by Fisher et al. (6) revealed that out of 153 women with ART pregnancies, 26 (17%) were admitted to the residential early parenting centers after delivery, indicating that this rate is 3.3 times greater than that of general population (5%). They also reported higher admission rate to such centers among women who were primiparou, did not receive adequate breastfeeding advice, had low level of confidence in caring for their children, had a postpartum mood disorder (PPMD), had an unsettled infant and received insufficient support. In our study, the findings showed that half of the ART women were primiparous and sought parenting advice and support from a wide range of resources, indicating that our findings are similar to those of Fisher et al. (6). Our study also offers important insights about the impact of a residential intervention on low parenting confidence of the mothers with a history of infertility. Furthermore, our study showed that the parenting programs in the residential units were effective and there were significant changes in the women’s parenting confidence after receiving parenting education. This was confirmed by a large effect size, indicating remarkable improvements in the parenting confidence of the mothers with a history of infertility. 

A study by Rowe and Fisher showed that 4-5 night stay in the Tweddle Child and Family Health Service resulted in significant improvements in the infant behaviours and maternal psychological functioning at one month after discharge, while these effects were sustained during the six-month follow-up period (23). In a study by Hayes et al. (24), they showed that the one-day intervention in a Melbourne metropolitan Day Stay Centre significantly improved parental confidence and infant’s behaviours at 6th week after discharge and lowered the levels of parental distress. Furthermore, parents reported that they were highly satisfied with the level of help they received from the nurses and social workers (85%), the involvement in individual care planning (83%) and the education provided in the parenting center (70%). 

Our findings are consistent with those of Rowe and Fisher and Hayes et al., indicating positive outcomes, such as improvements in the maternal mental health and child’s behaviour, bedtime routine and feeding habits after receiving intervention, training and supports in the residential early parenting centers. Phillips et al. (10) reported that a five-day stay in the Karitane RUs resulted in statistically significant improvements in sleeping time, unsettled behaviour, night-time waking and maternal depression and anxiety at one month and three months after discharge. In another study by Treyvaud et al. (25), they demonstrated remarkable improvements in the maternal parenting behaviour during parent-child interaction, depression, anxiety and stress. They also reported an improvement in maternal reports of child’s difficult behaviour. Also, more than half of the mothers participating in their study achieved 80% of their parenting goals after attending the program. Attending the residential parenting program results in less unsettled behaviour in the children and helps the children become more content during wakefulness and sleep for longer period of time both during day and at night. These changes in the children’s behaviour after attending the residential parenting centers may contribute to the improvement of the mothers’ parenting confidence and their better mental health status. Even one-day parenting programs have remarkable effects on the improvement of parenting skills and children’s unsettled behaviours (26). 

Results of our study showed that 14.8% of the mothers with a history of infertility reported symptoms of depression on the admission. Although the results of our study may not be generalized to the population due to the small sample size, these findings are in accordance with those of Klock (27) and Lee et al. (28), indicating that approximately 8-10% of the women with a history of infertility experienced symptoms of depression during postnatal period. 

In our study, due to only presence of singleton mothers, we were unable to compare the level of parenting confidence between mothers of singletons and mothers of twins/triplets. Nevertheless, Baor and Soskolne have indicated that poor parental adjustment and parenting stress are higher in the infertile women conceiving twins or triplets through the ART. As compared with the mothers conceiving twins spontaneously, mothers of the ART twins report poorer coping resources and higher levels of maternal stress during postnatal period (29). The risk of parenting stress is 22% in the mothers of ART triplets, compared with 5% of the mothers of ART singletons and 9% of the mothers of naturally conceived singletons (30). 

The present study had some limitations that need to be addressed. Firstly, due to use of previously recorded data of the client’s files, there was a lack of data on the paternal parenting confidence and depression. Secondly, due to lack of follow-up data on the maternal parenting confidence and depression post-discharge, this study failed to investigate the long-term effects of the intervention in this regard. Thirdly, this was a retrospective study in which we analyzed the previously recorded data of a small sample of mothers with a history of infertility who attended the Karitane RUs (27 women). Therefore, our results may not be generalized to the larger population of postpartum women with a history of infertility. Last but not least, due to the access issues, we were not able to evaluate the parenting confidence of the mothers with a history of infertility who were not admitted to the Karitane RUs. Future research with larger samples of women, from more varied backgrounds, with and without a history of infertility is needed to determine the extent to which the current findings are generalizable. 

## Conclusion

Results of this study demonstrated that 59.3% of the mothers with a history of infertility experienced low parenting confidence. These findings emphasized on the role of residential early parenting serviced in promoting the level of parenting confidence and highlighted the need for early recognition and referral of those who are at the risk to such centers. The mothers with a history of infertility need to be considered as a high-risk group and identified during the early stages of pregnancy and parenthood. Midwives, child and family health nurses, mental health clinicians and other health care providers that deal with the women who have babies after a period of infertility need to assess their mental health and parenting confidence before discharge from the hospital and follow them up to make sure they are supported throughout their journey. 
